# Cannabinoid Delivery Systems for Pain and Inflammation Treatment

**DOI:** 10.3390/molecules23102478

**Published:** 2018-09-27

**Authors:** Natascia Bruni, Carlo Della Pepa, Simonetta Oliaro-Bosso, Enrica Pessione, Daniela Gastaldi, Franco Dosio

**Affiliations:** 1 Istituto Farmaceutico Candioli, 10092 Beinasco, Italy; natascia.bruni@candioli.it; 2 Department of Drug Science and Technology, University of Turin, 10125 Turin, Italy; carlo.dellapepa@unito.it (C.D.P.); simona.oliaro@unito.it (S.O.-B.); 3 Department of Life Sciences and Systems Biology, University of Turin, 10123 Turin, Italy; enrica.pessione@unito.it; 4 Department of Molecular Biotechnology and Health Sciences, University of Turin, 10125 Turin, Italy; daniela.gastaldi@unito.it

**Keywords:** cannabinoids, delivery system, pain treatment, inflammation, cannabidiol, Δ^9^-tetrahydrocannabinol

## Abstract

There is a growing body of evidence to suggest that cannabinoids are beneficial for a range of clinical conditions, including pain, inflammation, epilepsy, sleep disorders, the symptoms of multiple sclerosis, anorexia, schizophrenia and other conditions. The transformation of cannabinoids from herbal preparations into highly regulated prescription drugs is therefore progressing rapidly. The development of such drugs requires well-controlled clinical trials to be carried out in order to objectively establish therapeutic efficacy, dose ranges and safety. The low oral bioavailability of cannabinoids has led to feasible methods of administration, such as the transdermal route, intranasal administration and transmucosal adsorption, being proposed. The highly lipophilic nature of cannabinoids means that they are seen as suitable candidates for advanced nanosized drug delivery systems, which can be applied via a range of routes. Nanotechnology-based drug delivery strategies have flourished in several therapeutic fields in recent years and numerous drugs have reached the market. This review explores the most recent developments, from preclinical to advanced clinical trials, in the cannabinoid delivery field, and focuses particularly on pain and inflammation treatment. Likely future directions are also considered and reported.

## 1. Introduction

Cannabis (*Cannabis sativa*) is a dioic plant that belongs to the Cannabaceae family (Magnoliopsida, Urticales). Knowledge of the medical and psychoactive properties of cannabis dates back to 4000 B.C. All of the different varieties of cannabis, including the one known as *Cannabis indica*, belong to the same species. All *C. sativa* plants produce active compounds, but each variety produces these compounds in different concentrations and proportions, which do not only depend on genomic background, but also on growing conditions and climate, meaning that they can be referred to as chemical varieties or chemovars, rather than strains [1]. Each chemovar contains varying concentrations of cannabinoids, a class of mono- to tetracyclic C21 (or C22) meroterpenoids. While more than 100 different cannabinoids can be isolated from *C. sativa*, the primary psychoactive compound is Δ^9^-tetrahydrocannabinol (THC), which was first isolated in its pure form by Gaoni and Mechoulam in 1964 [2]. Other pharmacologically important analogues are cannabidiol (CBD), cannabinol, cannabinoid acids, cannabigerol, and cannabivarins. In addition to cannabinoids, other components, such as the monoterpenoids myrcene, limonene, and pinene and the sesquiterpenoid β-caryophyllene, can also mediate the pharmacological effects of *C. sativa* [3].

Although phytocannabinoids have similar chemical structures, they can elicit different pharmacological actions. The identification of THC paved the way for the discovery, in 1988, of cannabinoid receptor type 1 (CB1) [4], and, later, of cannabinoid receptor type 2 (CB2) [5]. CB1 and CB2 belong to a family of seven transmembrane Guanosine Binding Protein-Coupled Receptors, are widely expressed and distinguished by their specific functions, localization and signalling mechanisms. They are one of the important endogenous lipid signalling pathways, named the ‘endocannabinoid system’, which consists of cannabinoid receptors, the endogenous ligands of cannabinoid receptors (endocannabinoids) and the enzymes that regulate the biosynthesis and inactivation of endocannabinoids. This lipid signalling system is involved in many important physiological functions in the central and peripheral nervous system and in the endocrine and immune systems [6,7]. 

The psychotropic effects of cannabis are principally mediated by CB1, which is widely distributed throughout the brain, but mainly in the frontal cortex, basal ganglia and cerebellum. CB1 is also present in several tissues and organs, including adipose tissue, the gastrointestinal tract, the spinal cord, the adrenal and thyroid glands, liver, reproductive organs and immune cells. The presence of CB1 receptors on chondrocytes and osteocytes, as well as evidence for their presence on fibroblast-like synoviocytes, makes CB1 particularly interesting in the study of rheumatic diseases [8]. CB1 activation inhibits adenylate cyclase and reduces cAMP levels and protein kinase A (PKA) activity, resulting in the activation of the A-type potassium channels and decreased cellular potassium levels [9].

CB2 is principally expressed in immune cells, but can also be found in various other cell types, including chondrocytes, osteocytes and fibroblasts, meaning that it can be considered the peripheral cannabinoid receptor. It is also present in some nervous tissues, such as dorsal root ganglia and microglial cells. CB2 shows 44% amino acid similarity with CB1, and similarly inhibits adenylate cyclase as well as activating mitogen-activated protein kinase. Moreover, CB2 activation can increase intracellular calcium levels via phospholipase C. While both CB1 and CB2 are coupled to G-proteins, the transduction pathways that they activate can be different, for example, in their interactions with ion channels [10]. The association of a particular variant of CB2, known as Q63R, with coeliac disease, immune thrombocytopenic purpura and juvenile idiopathic arthritis is particularly interesting for the field of autoimmune and rheumatic diseases [11]. 

Overall, seven different endogenous ligands have been identified as acting within the endocannabinoid system to date. The first two endocannabinoids are the derivatives of arachidonic acid *N*-arachidonoyl ethanolamide (anandamide) and 2-arachidonoyl glycerol [12]. A third endocannabinoid, 2-arachidonoyl glyceryl ether (noladin ether) was discovered in 2001. *N*-arachidonoyl dopamine, *O*-arachidonoyl-ethanolamide (virodhamine), docosatetraenoylethanol-amide, lysophosphatidylinositol and oleoylethanolamide have since been described as ligands of endocannabinoid receptors [7].

The endocannabinoid system’s contribution to the regulation of such a variety of processes makes phytocannabinoid pharmacological modulation a promising therapeutic strategy for many medical fields, including the studies of analgesic, neuroprotective, anti-inflammatory and antibacterial activity [13,14].

THC is the primary psychoactive component of cannabis and works primarily as a partial agonist of CB1 (Ki = 53 nM) and CB2 (Ki = 40 nM) receptors [15] and has well-known effects on pain, appetite enhancement, digestion, emotions and processes that are mediated through the endocannabinoid system [7]. Adverse psychoactive events can be caused by THC, depending on dose and previous patient tolerance. By contrast CBD, which is the major non-psychoactive phytocannabinoid component of *C. sativa*, has little affinity for these receptors, (Ki for human CB1 and CB2 of 1.5 and 0.37 µM, respectively), and acts as a partial antagonist CB1 and as a weak inverse CB2 agonist (Ki as antagonist of CP55940 from 4.2 ± 2.4 to 0.75 ± 0.3 µM in different human cell lines) [16].

In a recent paper, experiments based on the functional effects of CBD on PLCβ3, ERK, arrestin2 recruitment and CB1 internalization, show a negative allosteric modulation of CB1 at concentration below 1 µM [17]. 

Additionally, other non-CB1 receptor mechanisms of CBD have been proposed, among them its agonism at serotonin 1A receptor (or 5-TH1A), vanilloid receptor 1 (TRPV1) and adenosine A2A receptors [18,19]. The complex physiological and pharmacological mechanisms and interaction of CBD with the endocannabinoid system and other molecular targets are extensively reviewed by McPartland et al. [20]. These data may help explain some of the observed CBD effects including analgesic, anti-inflammatory, anti-anxiety and anti-psychotic activity [21]. The combination of THC and CBD with other phytocannabinoids and other components, such as terpenoids and flavonoids, in cannabis may have a synergistic effect on pain treatment [22,23].

## 2. Role of Cannabinoids in Inflammation and Pain 

**Figure 1:**
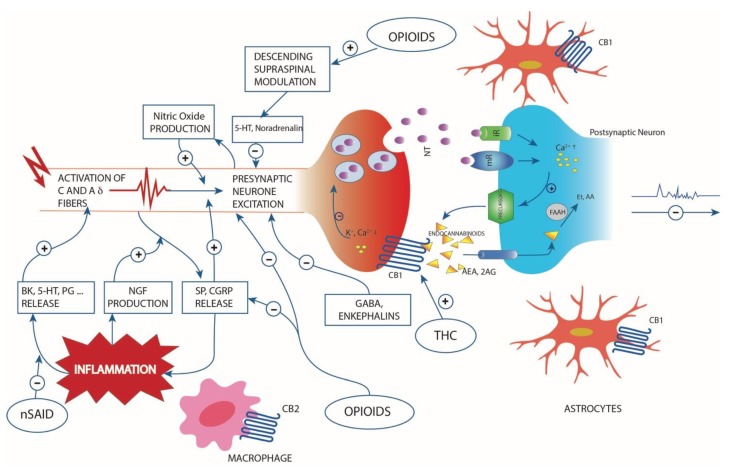
Simplified scheme representing the pathogenesis of pain following inflammatory disease or nociceptive stimulus, the cytokines involved in the process, the descending supraspinal modulation and the relive neurotransmitters and endocannabinoid retrograde signalling mediated synaptic transmission. Endocannabinoids are produced from postsynaptic terminals upon neuronal activation. Natural and synthetic cannabinoids act like the two major endocannabinoids shown in the scheme: 2-arachidonolglycerol (2-AG) and anandamide (AEA). Endocannabinoids readily cross the membrane and travel in a retrograde fashion to activate CB1 located in the presynaptic terminals. Activated CB1 will then inhibit neurotransmitter (NT) release through the suppression of calcium influx. NT can bind to ionotropic (iR) or metabotropic (mR) receptors. 2-AG is also able to activate CB1 located in astrocytes. Although endocannabinoid retrograde signalling is mainly mediated by 2-AG, AEA can activate presynaptic CB1 as well. Fatty acid amide hydrolase (FAAH) found in postsynaptic terminals is responsible for degrading AEA to AA and ethanolamine (Et). Inflammation lead to release of biochemical mediators (bradykinin (BK), serotonin (5-HT), prostaglandins (PG) etc.) and the up-regulation of pain mediator nerve growth factor (NGF). The substance P (SP) and calcitonin gene-related peptide (CGRP) vasoactive neuropeptides, released from sensory nerve, have also role in inflammation. The interaction with opioids, THC and nonsteroidal anti-inflammatory drugs are also represented.

Pain and inflammation are the body’s physiological responses to tissue injury, infection and genetic changes [24]. These responses can be divided into two phases: acute and chronic. The acute phase is the early, non-specific phase and is characterized by local vasodilatation, increased capillary permeability, the accumulation of fluid and blood proteins in the interstitial spaces, the migration of neutrophils out of the capillaries, and the release of inflammatory mediators (e.g., cytokines, lymphokines and histamine). Pain is produced by all these pro-inflammatory agents, that also lead to hyperalgesia through the activation of the corresponding receptors, which are expressed by nociceptive terminals (Figure 1). If the condition that causes the damage is not resolved, the inflammatory process progresses towards subacute/chronic inflammation, which is characterized by immunopathological changes, such as the infiltration of inflammatory cells, the overexpression of pro-inflammatory genes, the dysregulation of cellular signalling and the loss of barrier function. 

Chronic state of inflammation plays an important role in the onset of classic inflammatory diseases (e.g., arthritis) but also of various diseases, including cardiovascular and neurodegenerative diseases, diabetes, cancer, asthma. The suppression or inhibition of inflammatory/pro-inflammatory mediators using synthetic anti-inflammatory compounds (both steroidal and non-steroidal) is one of the major routes for the treatment of inflammatory disorders. However, several common side effects, including gastric irritation and ulceration, renal and hepatic failure, haemolytic anaemia, asthma exacerbation, skin rashes, are often associated with the use of synthetic anti-inflammatory drugs [25]. Increasing amounts of evidence demonstrate that the endocannabinoid system actively participates in the pathophysiology of osteoarthritis-associated joint pain. Production and release of endocannabinoids are mediated, during inflammatory-joint disease, by the generation of pro inflammatory cytokines (interferon [IFN]-c, interleukin (IL-12, IL-15, IL-17, IL-18), chemokines, chemical mediators, such as nitric oxide synthetase (NOS)-2, cyclooxygenase-2 (COX-2), matrix metalloproteinases (MMPs) and various other arachidonic acid metabolic by-products [7]. Overall, preclinical and clinical data support the potentially effective anti-inflammatory properties of endocannabinoid agonists that target CB2 receptors. 

The chronic pathological pain state, including neuropathic pain, is a leading health problem worldwide as it endures beyond the resolution of the pain source and can deeply impact quality of life [26]. Unlike physiological pain, in which tissue injury and/or inflammation can induce reversible adaptive changes in the sensory nervous system leading to protective sensitization, changes in sensitivity become persistent or chronic in neuropathic pain. Furthermore, the nervous system, peripheral or central, is injured in neuropathic pain. It is characterised by pain in the absence of a noxious stimulus and may be spontaneous in its temporal characteristics or be evoked by sensory stimuli (hyperalgesia and dynamic mechanical allodynia). For example, neuropathy is still among the most common diabetes complications, affecting up to 50% of patients, despite recent advances in treatment. There is no effective treatment with which to prevent or reverse neuropathic pain [27], thus current treatment is only directed at reducing symptoms. The treatment of chronic pain is still an unmet clinical need, where adequate pain relief is obtained using drugs with adverse effects on central nervous system side [28]. The quality of life of neuropathic pain patients is often aggravated by comorbidities such as sleep disorders, depression and anxiety compromise.

The finding of the endocannabinoid-mediated retrograde synaptic signalling pathway has opened up a new era, for cannabinoid research, including evaluations of their therapeutic use [29]. Selective CB2 agonists have shown considerable efficiency in a variety of neuropathic pain preclinical models, while increasing amounts of evidence, derived from clinical studies, have confirmed the potential of the cannabinoid system in affording benefits for patients with chronic pain and chronic inflammatory diseases (arthritis). Currently, patients with chronic arthritic and musculoskeletal pain are the most prevalent users of therapeutic cannabis products [30]. 

Preclinical studies have shown that cannabinoid receptor agonists block pain in various acute and chronic pain models and that inflammation is attenuated [31,32,33]. Both CB1 and CB2 receptor agonists demonstrate anti-nociceptive activity, whether used singly or in combination, with CB2 activity believed to affect microglial cells and thereby reduce neuro-inflammatory mechanisms [34,35]. The CB2 receptor is thought to be particularly important in central neuronal pain circuits, as agonist activity induces dopamine release in mid-brain areas, contributing to descending pain control and the placebo effect [36]. Inflammatory effects can either be modulated via the upregulation of cannabinoid receptor activity or increased production of endocannabinoids, providing an attenuation in joint destruction in preclinical models of inflammatory arthritis that mimic human rheumatoid arthritis [30,32]. Similarly, CB1 and CB2 receptor proteins and endocannabinoids are found in the human synovial tissue of patients with both rheumatoid arthritis and osteoarthritis [37]. 

Data from clinical trials on synthetic and plant-derived cannabis-based medicines have suggested that they are a promising approach for the management of chronic neuropathic pain of different origins [38,39,40]. It is also hypothesised that cannabis reduces the alterations in cognitive and autonomic processing that are present in chronic pain states [41]. The frontal-limbic distribution of CB receptors in the brain suggests that cannabis may preferentially target the affective qualities of pain [42]. Furthermore, cannabis may improve neuropathic pain reducing the low-grade inflammation consistent in the pathology [43]. Considering as a whole the problems of chronic neuropathic pain syndromes, which has a poorly understood pathogenesis, a complexity of symptoms and the lack of an optimal treatment, the potential of a therapeutic strategy centered on cannabinoid system appears really quite attractive. However, a range of adverse events (particularly somnolence or sedation, confusion, psychosis) may limit the clinical applications of therapeutics based on cannabis. Some current clinical guidelines and systematic reviews consider cannabis-based medicines as third- or fourth-line therapies for chronic neuropathic pain syndromes, for use when established therapies (e.g., anticonvulsants, antidepressants) have failed [44,45].

Beyond its effects on the inflammatory pathway, the endocannabinoid system also plays a fundamental role in neuronal development affecting axon and dendrite growth [46] and preclinical models have demonstrated that cannabinoid administration alters brain maturation in young animals and leads to neuropsychiatric consequences in adults [47]. Moreover, endocannabinoid system has also been accepted to play a significant role in the maintenance of gut homeostasis, and this is therefore, of particular interest in the management of inflammatory bowel diseases (i.e., Crohn’s disease and ulcerative colitis) that show increasing prevalence in Westernised countries [48].

## 3. Current Drug Dosage Forms and Novel Delivery Systems 

A modern pharmaceutical approach to administration may start from the use of the cannabis plant for medical use, and then move on to the development of quality controlled extracts, the complete evaluation of their analytical profiles, and studies to assess the delivery of the correct dosage for optimal therapeutic effect. Cannabinoids are highly lipophilic molecules (log P 6–7) with very low aqueous solubility (2–10 μg/mL) [49], that are susceptible to degradation, especially in solution, via the action of light and temperature as well as via auto-oxidation [50,51]. Formulation can thus play a crucial role in increasing the solubility and physicochemical stability of the drugs. Commonly used strategies in marketed products include salt formation (i.e., pH adjustment), cosolvency (e.g., ethanol, propylene glycol, PEG400 etc.), micellization (e.g., polysorbate 80, cremophor ELP etc.), (nano)-(micro)-emulsification, complexation (e.g., cyclodextrins), and encapsulation in lipid-based formulations (e.g., liposomes) and nanoparticles [52,53,54,55].

Various administration and delivery forms have been tested for therapeutic use. Cannabis products are commonly either inhaled by smoking/vaporization, or taken orally. The oromucosal, topical-transdermal and rectal routes are minor, but interesting, administration routes. The pharmacokinetics and dynamics of cannabinoids vary as a function of the route of administration with absorption showing the most variability of the principal pharmacokinetic steps. Absorption is affected both by intrinsic product lipophilicity and by inherent organ tissue differences (i.e., alveolar, dermal vs. gastric). A variety of factors, such as recent eating (for oral), depth of inhalation, how long breath is held for and vaporizer temperature (for inhalation) all affect cannabinoid absorption, which can vary from 20–30% for oral administration and up to 10–60% for inhalation. A reference review detailing the pharmacokinetic and pharmacodynamic aspects of cannabinoids has been written by Grotenhermen [49]. The following sections explore the principal administration routes for cannabinoids, available products and the principal strategies (extracted from scientific literature and patents) that can be applied to improve cannabinoid efficacy and stability. Treatment indications and their level of evidence are also reported while the principal characteristics of the formulations have been summarized in Table 1.

### 3.1. Oral Route 

The primary advantages displayed by the oral administration of cannabinoids include the existence of pharmaceutical-grade compounds, standardized concentrations/doses and a non-complicated administration route. Oils and capsules currently allow for more convenient and accurate dosing than juices or teas from the raw plant. Nevertheless, absorption is slow, erratic and variable. Maximal plasma concentrations are usually achieved after 60–120 min, although this can take even longer (up to 6 h) and can be delayed. Furthermore, metabolism produces psychoactive metabolites. Extensive first-pass liver metabolism further reduces the oral bioavailability of THC, while effect duration varies from 8 to 20 h. Numerous (nearly 100) metabolites have been identified as being produced, primarily in the liver and, to a lesser degree, in other tissues, such as the heart and lungs [49].

There are three oral, and one oromucosal, cannabinoid pharmaceutical preparations that are currently available. 

Dronabinol (Marinol^®^ from Abbvie Inc., Chicago, IL, USA) is a semi-synthetic form of THC, which is available in capsule form and as a solution, that has been approved by the FDA for appetite stimulation and the treatment of chemotherapy-induced nausea in patients with AIDS. Oh et al. have published a PK study that compares the oral solution and capsule forms of dronabinol under fasting and fed conditions. The solution formulation showed lower inter-individual absorption variability than the capsule formulation, especially in fed conditions, and this fact may be an important consideration in the selection of an appropriate dronabinol product for patients [56]. Dronabinol exerted a modest, but clinically relevant, analgesic effect on central pain in the pain treatment of patients with multiple sclerosis. Although the proportion of patients that showed adverse reactions was higher in dronabinol-treated than in placebo-treated patients, it decreased over the drug’s long-term use [57,58]. 

**Figure 2:**
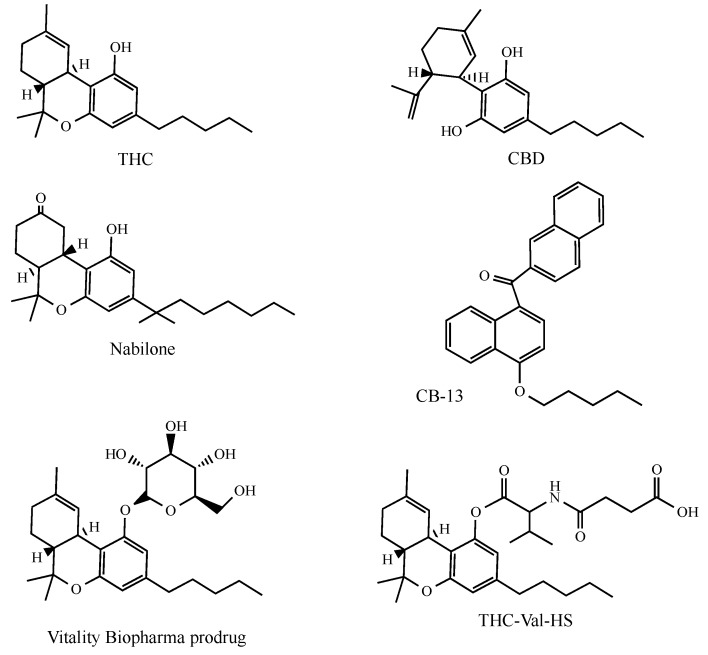
The structures of the principal cannabinoids described in the text.

Nabilone (Cesamet^TM^ from Bausch Health Co., Laval, QC, Canada) is a synthetic cannabinoid derivative that differs structurally from THC as its C-ring is saturated and contains a C-9 ketone group (Figure 2). Nabilone is available, in a polyvinylpyrrolidone carrier, as a capsule (1 mg of drug). It displays antiemetic properties and is used for the control of the nausea and vomiting associated with cancer chemotherapy in patients who have failed to respond adequately to conventional antiemetics [59]. 

Nabilone has higher bioavailability than dronabinol (95% vs. 10–20%) and presents a higher duration of action. Nabilone has recently proven itself to be a suitable and safe therapeutic option with which to aid in the treatment of cancer patients diagnosed with anorexia. An enriched enrolment, randomised withdrawal design trial (26 patients) assessed the efficacy of nabilone, in the treatment of diabetic peripheral neuropathic pain [60]. Nabilone has an interesting range of applications (e.g., quality of life in lung cancer patients) although larger trials are still necessary if more robust conclusions are to be drawn [61].

Epidiolex (from GW Pharmaceuticals plc, Cambridge, UK), is a liquid formulation of a CBD solution that has recently been approved in the US as an adjuvant treatment in Dravet syndrome, Lennox-Gastaut syndrome and severe myoclonic epilepsy in infancy. Results from double-blind, placebo controlled trials have recently been published [62,63,64].

Furthermore, other improved oral-dosage formulations and therapeutic applications have been presented in a number of patents. Clinical considerations of the oral administration of a solid-dosage, CBD-containing form for the treatment of inflammatory bowel disease have been published in a patent by Robson (GW patent) [65]. A small cohort of patients (8 patients) reported an improvement in Crohn’s disease. Furthermore, oral administration also led to another small cohort of patients being able to reduce steroid dose when treating inflammatory and autoimmune diseases [66]. Based on this research, a CBD therapeutic formulation is being developed by Kalytera Therapeutics (Novato, CA, USA) for the prevention and treatment of graft-versus-host disease. Kalytera initiated a randomised, open-label, dose-response and comparator-controlled phase IIb trial in December 2017 to evaluate the pharmacokinetic profile, safety and efficacy of multiple doses of CBD for the prevention of graft-versus-host-disease following allergenic haematopoietic cell transplantation (NCT02478424).

The manufacture, specifications, pharmaceutical tests and preliminary pharmacokinetics of CBD-containing, compressed tablets and granulates for peroral delivery have been reported in a patent by De Vries et al. [67]. 

Self-emulsifying drug delivery systems (SEDDS) can be significant in improving the dissolution, stability and bioavailability of THC and other cannabinoids. SEDDS, which are isotropic mixtures of oils, surfactants, solvents and co-solvents/surfactants, can be used in the design of formulations to improve the oral absorption of highly lipophilic drug compounds [68]. Murty et al. have described self-emulsifying drug delivery systems for *per os* administration in a number of patents, with the aim of improving the dissolution, stability and bioavailability of THC and other cannabinoids [69,70,71]. The solubility of the selected drug, in oils (soybean and sesame oils, oleic acid) and surfactants (Oleoyl polyoxyl-6 glycerides, medium-chain mono- and di-glycerides and propylene glycol esters, PEG hydrogenated castor oil) was assessed.

A CBD therapeutic formulation is being developed by Kalytera Therapeutics for the prevention and treatment of graft-versus-host disease. Kalytera initiated a randomised, open-label, dose-response and comparator-controlled phase IIb trial in December 2017 to evaluate the pharmacokinetic profile, safety and efficacy of multiple doses of CBD for the prevention of graft-versus-host-disease following allergenic haematopoietic cell transplantation (NCT02478424).

Vitality Biopharma (Los Angeles, CA, USA) have proposed an invention that has led to several cannabinoid glycoside prodrugs (cannabosides) being obtained and characterized [72,73] (Figure 2). This method grants the gastro-intestinal targeting of THC, while avoiding narcotic effects. Vitality Biopharma have released data from independent clinical trial case studies which demonstrate that cannabinoids induced the remission of drug-resistant inflammatory bowel disease after eight weeks of treatment (Vitality Biopharma web site). 

### 3.2. Administration through Mucosa 

Drugs, such as cannabinoids, that are metabolized by liver and gut enzymes (first-pass hepatic metabolism), have specific pharmacokinetic requirements, demonstrate poor gastrointestinal permeability and cause irritation and therefore require alternatives to systemic oral delivery. Transdermal, nasal, inhaled-pulmonary and oral transmucosal delivery formulations enable drug uptake directly into the blood, thereby eliminating first-pass metabolism.

The development of the transmucosal dosage form has provided a non-invasive method of administration that has proven itself to be significantly superior to oral dosage in the relief of pain (e.g., oral morphine vs transmucosal fentanyl) [74].

Nabiximols (Sativex^®^ from GW Pharmaceuticals plc), is an oromucosal spray that contains a roughly 1:1 ratio of THC and CBD, as well as specific minor cannabinoids and other non-cannabinoid components (β-caryophyllene). It is administered at a dose that is equivalent to 2.7 mg THC and 2.5 mg CBD in each 100 μL ethanol spray. THC and CBD may reciprocally interact either by interfering with each other’s pharmacokinetics, or, at the cellular level, within the complex endocannabinoid signalling network. However, a study involving nine cannabis smokers reported that no significant pharmacokinetic differences were found in the similar oral THC and Sativex^®^ doses that were administered [75]. Furthermore, studies have suggested that the adverse effects of THC can be antagonized by CBD [76].

Nabiximols is used as an adjunctive treatment for the symptomatic relief of moderate to severe multiple sclerosis-caused spasticity in adults who have not responded adequately to other therapies, and who show clinically significant improvements in spasticity-related symptoms during an initial therapy trial. It may also be of benefit as an adjunctive analgesic treatment for the symptomatic relief of neuropathic pain in adult patients with multiple sclerosis. This same preparation is also used as an adjunctive analgesic treatment in adult patients with advanced cancer who have moderate to severe pain during the highest tolerated dose of strong opioid therapy for persistent background pain [77]. Although not superior to placebo in terms of the primary efficacy endpoint, nabiximols provided multiple secondary endpoint benefits, particularly in patients with advanced cancer who receive a lower opioid dose, such as individuals with early intolerance to opioid therapy.

Nabiximols has now received marketing authorization in EU countries for the treatment of spasticity and FDA investigational new drug (IND) status for the treatment of cancer pain. Some clinical trials into the use of Sativex for the treatment of neuropathic pain in multiple sclerosis patients have been successful [78], leading to the drug gaining approval in Israel and Canada. However, further work is still required to define the best responder profile for nabiximols and to explore its full potential in this field is still required. 

Transmucosal formulations of CBD with Poloxamer 407, carboxymethyl cellulose and starch have been reported by Temtsin-Krayz et al. Nanoscale-range powders have been produced using the spray drier technique. Crossover bioavailability comparisons of this formulation and Sativex have also been reported [79].

A controlled-release chewing gum, made up of a (1:1) combination of CBD and THC, which provides oromucosal adsorption is being developed by Axim Biotech. Inc., (New York, NY, USA). The product is currently in clinical trials for the treatment of several diseases (pain, multiple sclerosis-associated spasticity, Parkinson’s disease, post-herpetic neuralgia, dementia etc.) [80]. More recently, Axim have also proposed chewing gums that are formulated to provide the controlled release of microencapsulated cannabinoids, opioid agonists and/or opioid antagonists during mastication [81].

The intranasal mode of administration (in which drugs are insufflated through the nose) has several advantages; the nasal cavity is covered by a thin mucosa that is well vascularised, meaning that a drug can be transferred quickly across the single epithelial cell layer directly into systemic blood circulation and avoid first-pass hepatic and intestinal metabolism, producing a fast effect. Bypassing the oral route may be more acceptable for patients who experience nausea, vomiting, oral mucositis and impaired gastrointestinal function. Furthermore, intranasal delivery is superior to iv injection because it is a non-invasive pain-free treatment that can improve patient compliance. The development of a nasal formulation of CBD could potentially aid in the treatment possible breakthrough pain and nausea attacks. 

Paudel et al. have prepared a variety of formulations (CBD in PEG 400 alone and CBD in a 50:35:15 (*v*/*v*) PEG: saline:ethanol solvent system both with and without the following permeation enhancers: 1% sodium glycocholate or 1% dimethyl-beta-cyclodextrin) for the investigation of the intranasal permeation of CBD in an anesthetized rat nasal absorption model [82]. The intranasal application of CBD formulations resulted in the significant and relatively rapid absorption of CBD from the nasal cavity. The nasal absorption of CBD from all the formulations was rapid (T_max_ ≤ 10 min), while the absolute CBD bioavailability achieved by the different nasal formulations was in the 34–46% range. Bioavailability decreased when the PEG content of the formulation was lowered from 100% to 50%, while the addition of permeation enhancers did not lead to AUC enhancements.

Bryson has described both semi-solid and liquid nasally administered cannabinoid compositions and a device to provide precise nasal administration [83]. A range of different formulations were described in the patent.

### 3.3. Pulmonary Administration

The intrapulmonary administration of cannabinoids is regarded as an effective mode of delivery as it results in the fast onset of action and high systemic bioavailability. Cannabis-related effects generally begin within a few minutes of the first inhalation (smoked or vaporized) and these effects can increase [84]. A peak value is reached after 10 min, and is maintained at a steady state for 3–5 h, which is in accordance with the plasma levels of THC [85]. Interestingly, the PK profile of inhaled cannabis is similar to that of intravenously administered THC, although it displays a lower AUC. The PK profile of CBD is very similar to that of THC, whether it is administered orally, intravenously or inhaled. These pharmacokinetics (rapid onset, short time peak effect and intermediate lasting effects) occur because first passage metabolism is avoided and are thus virtually impossible to replicate with the oral administration of cannabis or cannabinoids. The major limitation of inhaling is the variability in inter-patient efficiency that is caused by differences in inhalation techniques, respiratory tract irritation during inhalation, etc. In fact, improved methods with which to standardise dosage have been proposed for these very reasons. 

A protocol to deliver CBD and THC via vaporisation has been described by Solowij et al. Crystalline-form CBD (preliminary experiments), and ethanolic solutions of CBD (4 or 200 mg) and THC (4 or 8 mg) were separately loaded onto a vaporiser filling chamber via a liquid pad (a removable disc made of tightly packed stainless steel wire mesh) as supplied by the manufacturer of the Volcano^®^ vaporizer device [86].

A system, which combines method, devices and systems, for the controlled pulmonary delivery of active agents has also been reported; a metered dose inhaler to vaporize precise amount of agent (cannabinoids or other plant oils), a system for the evaluation of the PK value obtained after one or two puffs and an interface for the control of the profile of the drug administered have been provided by Davidson et al. [87].

Several patents have presented systems for vaporisation and nebulisation, from a variety of containers [88], at a selected temperature to form a precise amount of vapour with THC and CBD [89]. Improved drug-delivery devices that can separate and release active cannabis substances have been disclosed in another patent [90]; drug delivery cartridges, which include a substrate coated with at least one of either THC or CBD, are configured to allow for the passage of air through the cartridge to volatilise the agent for inhalation by a user.

### 3.4. Topical and Transdermal Route

Transdermal administration delivers drugs through the skin via patches or other delivery systems. Although comparable to oral-dosage forms in term of efficacy, transdermal patches provide numerous advantages. Transdermal administration avoids the first-pass metabolism effect that is associated with the oral route and thus improves drug bioavailability. Furthermore, transdermal administration allows a steady infusion of a drug to be delivered over a prolonged period of time, while also minimising the adverse effects of higher drug peak concentrations, which can improve patient adherence. Topical administration is potentially ideal for localised symptoms, such as those found in dermatological conditions and arthritis but also in peripheral neuropathic pain for which capsaicin patches have been proposed as a second line treatment after high quality of evidence was provided [91]. However, there are some disadvantages to consider, such as the possibility of local irritation and the low skin penetration of drugs with a hydrophilic structure. Indeed, drugs that are slightly lipophilic (log P 1–4), have a molecular mass of less than 500 Da and that show efficacy at low dosage (less than 10 mg/day for transdermal administration) are ideal for administration via this route. Enhancers may also be also added to transdermal formulations to increase the penetration of permeants by disrupting the structure of the skin’s outer layer, i.e., the stratum corneum, and increasing penetrant solubility.

The evaluation stages for the transdermal administration of cannabinoids range from early preclinical phases and mouse models, to self-initiated topical use and randomized, double-blind controlled studies. 

The topical anti-inflammatory activity of phytocannabinoids in a roton oil mouse ear dermatitis assay has been described by Tubaro et al. [92], while preclinical evaluations of the transdermal administration of CBD, via gel application, has been further tested on a rat complete Freund’s adjuvant-induced monoarthritic knee joint model [93]. In this latter study, CBD was found to demonstrate therapeutic potential for the relief of arthritic pain-related behaviour and to exert an anti-inflammation effect without any evident high-brain-center psychoactive effects. Results showed that a dose of 6.2 mg/day reduced knee-joint swelling and that increasing the dose to 62 mg/day failed to yield additional improvements. The transdermal administration of CBD has also been observed to provide better absorption than the oral administration route in same arthritic model [30].

Ethosomal carriers are mainly composed of phospholipids, (phosphatidylcholine, phosphatidylserine, phosphatidic acid), with a high concentration of ethanol and water [94]. An ethosomal formulation for CBD, which consisted of 3% CBD and ethanol in a carbomer gel, has been prepared by Lodzki et al. [95], and its anti-inflammatory effect was tested on carrageenan-induced aseptic paw oedema in a mouse model. The results demonstrated that the carrageenan-induced development of an oedema was only prevented in its entirety in the CBD-pretreated group of mice. The in vivo occluded application of CBD ethosomes to the abdominal skin of nude mice resulted in high accumulation of the drug in the skin and the underlying muscle.

A topical transdermal gel containing a proprietary and patent-protected CBD formulation is being developed by Zynerba Pharmaceuticals (Devon, PA, USA) and is currently in clinical development for the treatment of epilepsy, developmental and epileptic encephalopathy, fragile-X syndrome and osteoarthritis [96,97,98]. The gel is designed to be applied once or twice daily. Permeation profiles of a range of formulations have also been reported [99].

A particularly interesting, although anecdotal, result has recently been published by Chelliah et al., who described the benefits that CBD provided as anti-inflammatory agent in three patients affected by epidermiolysis bullosa. Paediatric patients benefited from the use of topical CBD (applied as an oil, cream and spray by their parents) leading to a reduction in pain and blistering as well as rapid wound healing [100]. There were no adverse effects reported, either by the patients or their families, of this topical use of CBD.

The release of cannabinoids from a microneedle formulation that is administered transdermally has been reported by Brooke [101], while a patent by Weimann has more recently focused on CBD delivery [102]. In this latter work, a solution of CBD 10% in ethanol with modified cellulose gave a thixotropic preparation that was placed in a reservoir. Diffusion through the skin occurs and is measured using hydrophilic and hydrophobic membranes. A monolithic version, also containing penetration enhancers (oleic acid and propylene glycol), was also prepared for comparison purposes. Linear release was observed for 24 h and cumulative amounts exceeded 200 µg/cm^2^.

A range of patents for the topical administration of CBD, mixed with other well-known anti-inflammatory phyto-derived products, will also be summarised here, as will their adsorption and effect on pain relief. 

Siukus has presented an oleo gel composition made up of non-psychoactive *Cannabis sativa* components for the treatment and/or reduction of deep tissue joint and muscle inflammation caused by mechanical skeletal muscle trauma and arthritis/osteoarthritis. The oleo gel composition is based on phytocannabinoids (2% of total mass) mixed with an extract of *Olea europaea* (Olive) (82%), *Mentha arvensis* leaf oil (0.5%), and anhydrous colloidal silica (8.2%) [103]. Preclinical evidence was reported. 

The same author has more recently published a patent that describes a topical composition made up of an essential combination of synergistically acting phytoactive materials and non-psychotropic phytocannabinoids in combination with a *Calendula* flower extract (*Calendula officinalis* L.) and the base formulation to provide anti-inflammation, anti-oxidation, emollient and bactericidal activity [104].

Jackson et al. [105] have proposed a topical administration of CBD with silicon fluids, coupled with hyaluronic acid. This system is claimed to enhance application methods and improve absorption into the skin to help ease pain. 

The use of cannabinoids, in combination with odorous volatile compounds and emu oil has also been proposed as a method to improve the effectiveness of cannabinoid transdermal delivery to areas in the hypodermis [106].

The application of CBD with argan oil for the treatment of the pain and swelling associated with inflammation, in arthritic and rheumatic diseases, has been described by Shemanky et al. [107]. Gel, cream and emulsion formulations were tested. 

Improved anti-inflammatory effects can be obtained from a composition containing boswellic acids, either isolated from Boswellia family plants (Buseraceae) or in the form of an extract, and either CBD or a *Cannabis sativa* extract [108].

In order to complete this overview of topical CBD, we should note that CBD exerts interesting sebostatic and anti-inflammatory effects on human sebocytes [109], (data obtained from in vitro evaluations). Indeed, CBD has been shown to inhibit the proliferation of hyperproliferative keratinocytes [54], and to possess remarkable antibacterial activity [55]. The authors also demonstrated the potent local activity of CBD as an anti-acne agent. Furthermore, its high lipophilicity means that CBD is expected to preferentially enter the skin via the transfollicular route and to accumulate in the sebaceous gland. 

Finally, the topical (ocular) administration of THC prodrugs has been proposed as a treatment to reduce intraocular pressure in glaucoma [110]. THC appears to be especially attractive in this case as, in addition to its intra ocular lowering activity, the presence of cannabinoid receptors in ocular tissues has recently been confirmed [111]. Hydrophilic THC prodrugs have been obtained by linkage with valine, with dipeptides and amino acid-dicarboxylic esters (Figure 2). Among them the best corneal permeability and intraocular pressure-lowering activity shown by these prodrugs were observed in the THC-Val-HS emulsion and micellar solution formulations.

### 3.5. Nano-Technological Approaches

Pharmaceutical nanotechnology is widely used in drug delivery as it can develop devices that are specifically adapted to improving the therapeutic efficacy of bioactive molecules. Indeed, nanocarriers, such as nanoemulsions, dendrimers, micelles, liposomes, solid lipid nanoparticles and nanoparticles of biodegradable polymers for controlled, sustained and targeted drug delivery, are popular and present possible alternatives to traditional formulation approaches. Nanovectors for drug delivery potentially offer a number of advantages: more efficient delivery of highly lipophilic drugs at high doses, protection from aggressive environments (e.g., acidic pH in the digestive tract), as well as targeted and controlled delivery to achieve precise administration to a specific tissue over a determined period of time (e.g., pegylation [113], coating with polysaccharides [114], etc.). Even though the use of nanocarriers as drug-delivery systems offers many advantages, there are still some drawbacks that need to be addressed: instability during blood circulation, low renal clearance, limited accumulation in specific tissues and low uptake by target cells. Physico-chemical aspects, such as surface charge, size, shape and deformability, modulate uptake and interactions with host cells as well as influencing uptake by immune cells, the subsequent immune responses and nanovector biodegradation [115]. An interesting work on the limitations, opportunities and concerns in this field has recently been published by Park [116]. Significant research effort has been dedicated to the development of nanocarriers for the treatment of cancer, neurological diseases, cardiovascular diseases and use as antimicrobial agents, for which the principal route is systemic administration. 

Their high lipophilicity and low stability (degradation via the effects of temperature, light and auto-oxidation can occur) mean that cannabinoids benefit greatly from nanotechnology approaches [51]. Indeed, recent years have seen micellar, liposomal and nanosized formulations being proposed for use in topical and systemic preparations. A brief description of the approaches presented in patents and in the literature, follows, while principal formulation data are reported in Table 2. 

#### 3.5.1. Lipid Carriers

Although liposomes are one of the most frequently studied and used market-approved drug delivery systems [55], only a few patents involving cannabinoids have been published. The main disadvantage for liposomes in the encapsulation of lipophilic compounds is their reduced ability to locate such compounds in their phospholipid bilayer. Low encapsulation efficiency, or drug loading (ratio of encapsulated drug/sum of all components), is normally obtained for this reason. Rapid bioavailability and onset in the pulmonary administration of loaded-THC liposomes has been reported by Hung [117]. The formulation was composed of dipalmitoylphosphatidylcholine and cholesterol, giving liposomes with an average size of 300–500 nm containing 0.3 mg/mL THC. Pharmacokinetic data described slow and prolonged release that continued for more than 5 h after administration. 

Micellar and liposomal preparations have also been proposed by Winniki et al. [118]. Micelles of 1 μm diameter were obtained via solvent injection in water and rapid solvent removal, while liposomes were produced using phosphatidylcholine ~52%, phosphatidylethanolamine 20%, phospholipids 26% and other compounds in a 2% mixture, via film hydration and solvent injection, ultrasonication and calcium alginate encapsulated liposomal suspension. Stability ranged from a few days (micelles) to several months (liposomes). 

A nano-technology platform proposed by Medlab Clinical (Sydney, NSW, Australia), named NanoCelle^TM^, that is made up of micelles obtained by mixing oils, glycerol and non-ionic surfactants is currently undergoing advanced trails. Micelles of nanometer size (less than 100 nm) and positive average Z potential have been observed to deliver lipophilic molecules (vitamin D3, statins, testosterone propionate, CBD) for absorption across the oral buccal mucosa, bypassing the gastrointestinal tract. Early research into their use in the treatment of pain is underway in Australia [119,120].

Lipid nanoparticles in a solid particle matrix are produced from oil/water emulsions by simply replacing the liquid lipid (oil) with a solid lipid, i.e., one that is solid at body temperature. First generation analogues, produced from a solid lipid only, are named solid lipid nanoparticles. The second generation of nanostructured lipid carrier (NLC) particles are produced from a blend of a solid lipid and a liquid lipid, in which the partially crystallized lipid particles, with mean radii ≤ 100 nm, are dispersed in an aqueous phase containing one or more emulsifiers [121]. NLC can be considered suitable carrier systems for THC and CBD because they make use of solid particle matrices instead of fluid matrices, such as emulsions and liposomes, meaning that NLC can better host substances and protect them from degradation. The solid particle matrix is also able to slow the diffusion of THC from inside the particle to the particle surface.

Esposito et al. have described the development of a method to encapsulate cannabinoid drugs (precisely the inverse agonist of the CB1 receptor (AM251 and Rimonabant) and the URB597 fatty acid amide hydrolase inhibitor) in NLC [122]. In this circumstance, the lipid phase was composed of tristearin/tricaprylin 2:1 while Poloxamer 188 was added to the water phase. Nanoparticles of around 100 nm with high encapsulation efficiency were obtained. 

NLC have recently been proposed for administration as a dosage form for nasal delivery. Nanospheres of 200 nm diameter, composed of either cetyl palmitate or glyceryl dibehenate and loaded with THC were obtained. In vitro mucoadhesion evaluations have revealed that cationic NLC formulations (obtained via the addition of cetylpyridinium chloride) should have high mucoadhesiveness properties [123]. The solid matrix of the NLC was found to have a stabilizing effect on THC. Indeed, 91% of the THC was unaltered after 6 months storage at 4 °C. About 1.7 mg THC is administered with one spray of the 0.25% THC-loaded NLC formulation in each nostril. This amount was close to the THC amounts obtained from the oromucosal formulation in a study by Johnson et al. [124]. 

Lipid nanoparticle formulations have been also reported, by Duran-Lobato et al. [125], to incorporate and deliver CB-13, a cannabinoid drug that acts as a potent CB1/CB2 receptor agonist, and show therapeutic potential. Nanoparticles composed of either glyceryl dibehenate or glyceryl palmitostearate and stabilized with two different surfactants (polysorbate 20 and sodium deoxycholate), were produced using the emulsification-solvent evaporation method. The best formulation in terms of size (120 nm) and polydispersity was obtained using glyceryl palmitostearate as the lipid matrix, which was effective, in the presence of lecithin, in the preparation of cannabinoid-loaded particles with high EE (around 99%) and stability upon storage at 4 °C. In vitro biocompatibility was assessed and demonstrated that that this type of formulation is safe. Furthermore, neither free CB-13 nor LNP produced cytotoxic effects in three cell lines at the tested dose (250 μg/mL of each LNP formulation for 24 h). This formulation was also stable under intestinal conditions, seemingly making it suitable for the oral delivery of CB-13.

Formulations that are based on self-(nano)emulsifying drug delivery technology (SEDDS) have been proposed as a means of improving the oral bioavailability of drugs that show poor aqueous solubility [126]. The base formulation, which is an isotropic mixture of an active compound in combination with lipids, surfactants and a co-solvent, has been called a pro-nano-liposphere (PNL) pre-concentrate and is ingested as a soft gelatine capsule. When it reaches the aqueous phase of the gastrointestinal tract, the PNL spontaneously forms a drug-encapsulated oil/water micro-emulsion with a particle diameter of less than 60 nm. The clinical usefulness of SEDDS, which stems from their ability to increase the solubility and oral bioavailability of poorly soluble drugs, have led to them attracting considerable interest [127]. Products, such as Sandimmune^®^ Neoral (cyclosporin A), Fortovase^®^ (saquinavir) and Norvir^®^ (ritonavir), have confirmed the value of this approach [128]. PTL401 is the proprietary PNL-based formulation of THC and CBD. The PTL401 formulation is composed of THC-CBD (1:1) in a formulation with polysorbate 20, sorbitan monooleate 80, polyoxyethylene hydrogenated castor oil 40, glyceryl tridecanoate, lecithin and ethyl lactate [129,130]. The CBD-THC PNL formulation also allows absorption enhancers, such as curcumin, resveratrol and piperine, to be incorporated. PK evaluations in a rat model have indicated that only piperine enhanced the oral bioavailability of CBD in-vivo [130]. Moreover, the enhanced oral bioavailability can be attributed to the inhibition of intestinal processes, rather than those of hepatic first-pass metabolism, while additional increases in the AUC of CBD prove that piperine-PNL also has an effect on phase II, and not on just phase I, metabolism. THC-CBD-piperine-PNL demonstrated higher absorption rates than Sativex^®^ in human volunteers, with peak values of 1 h for both THC and CBD, versus 3 h for THC and 2 h for CBD, respectively. Furthermore, the incidence and severity of reported adverse events were similar in both groups [131,132]. Nevertheless, regarding the role of piperine, it is important to remember that it is able to alter the metabolism of many drugs, being a cytochrome and glucuronyl transferase inhibitor. In addition, piperine demonstrates non-negligible toxicity (it is Generally Recognized as Safe only up to 10 mg/day).

Micro and nanoemulsions of active annabis ingredients (cannabinoids and terpenes) have also been presented in a patent [133], which proposes rectal-vaginalC and solid oral dosage forms. 

A proprietary CBD nanotherapeutic formulation (CTX01) for subcutaneous administration is being developed by Cardiol Therapeutics (Oakville, ON, Canada) the treatment of heart failure with preserved ejection fraction. Preclinical studies are currently under way (Cardiol web site) [134]. 

#### 3.5.2. Polymeric Carriers

Polymers have played an integral role in the advancement of drug delivery technology and this field has grown tremendously. Polymers are currently used in pharmaceutical formulations and show a wide range of safety and biodegradation variables. Developments in responsive polymers, polymer therapeutics and advanced systems for molecular recognition or for the intracellular delivery of novel therapeutics have more recently appeared [135,136]. Polymeric drug delivery systems are able to protect drugs from degradation and control drug release.

The poly (lactic-co-glycolic acid) (PLGA) polymer is one of the most commonly used materials for the encapsulation of drugs, as it is mechanically strong, hydrophobic, biocompatible and degrades into toxicologically acceptable products that are eliminated from the body.

PLGA nanoparticles, loaded with CB-13 for oral delivery, have been coated with a variety of agents (chitosan, Eudragit RS, vitamin E and lecithin) [137]. The nanoparticles exhibited particle sizes of 253–344 nm and high entrapment efficiency values (around 85%). Higher release rates were obtained with vitamin E and lecithin surface modification. Biodistribution evaluations revealed that none of the proposed surface modifications prevented the opsonisation process (liver and spleen uptake). Nonetheless, CB-13, which is highly lipophilic and displays low water solubility, can be absorbed well when it is included in these surface-modified polymeric carriers. 

Biocompatible polymer PLGA was preferred by Martin-Banderas for the preparation of THC-loaded nanoparticles for use as an anticancer agent [138]. Nanoparticles, with sizes ranging from 290–800 nm, were obtained with PEG, chitosan and PEG-chitosan being used as coating agents. Encapsulation efficiency and drug loading (around 96% and 4.8%, respectively) were not affected by the type of coating used and sustained drug release, of up to 10 days, was obtained. Surface modification with PEG reduced protein adsorption and thus, most likely, the in vivo opsonisation processes.

Poly-ε-caprolactone (PCL) is another polymer that is widely used in drug delivery systems. This is a biocompatible, biodegradable, FDA-approved, semi-crystalline aliphatic polyester that degrades slowly. Hernán Pérez de la Ossa has developed a formulation in which CBD is loaded into PCL particles. Spherical microparticles, with a size range of 20–50 μm and high entrapment efficiency (around 100%), were obtained. CBD was slowly released over within ten days when dissolved in the polymeric matrix of the microspheres in an in vitro test [139].

## 4. Critical Overview of Clinical Studies

Contrasting the abundance of public domain comment on the therapeutic effects of cannabinoids is the fact that there has only been a limited number of rigorous clinical studies on the topic, due to the illegal status of cannabinoids in most countries. Nevertheless, the licensing of Cannabis-based medicines, including herbal Cannabis for people with chronic (neuropathic) pain, is scheduled to occur in some countries and has already happened in Canada, Germany and Israel. Heated debate as to the true efficacy and side effects of Cannabis products and derivatives is therefore on-going. In 2017, the Health and Medicine Division of the US National Academies concluded that there is substantial evidence to support the claim that cannabis is effective for the treatment of chronic pain (cannabis), especially neuropathic pain in adults, for use as antiemetics in the treatment of chemotherapy-induced nausea and vomiting (oral cannabinoids), and as a means to improve patient-reported multiple sclerosis spasticity symptoms (oral cannabinoids) [140]. Nevertheless, only in recent years have a significant number of systematic reviews and meta-analyses evaluated the effects of all cannabinoids in all diseases and focused on cannabinoid use for chronic pain. Whiting et al. selected 79 trials and concluded that there was moderate-quality evidence to support the use of cannabinoids for the treatment of chronic pain and spasticity, while there was low-quality evidence for improvements in nausea and vomiting due to chemotherapy, weight gain in HIV, sleep disorders, and Tourette syndrome. Cannabinoids were also associated with an increased risk of short-term side effects [141]. Nugent et al. selected 29 chronic pain trials and suggested that there is some, limited evidence to indicate that cannabis is able to alleviate neuropathic pain in some patients, but also that insufficient evidence exists in other types of chronic pain [142]. Furthermore, Mücke et al. have also declined to share in the optimistic conclusions that cannabis-based medicines are effective, well-tolerated and safe in the treatment of chronic neuropathic pain, due to a lack of high-quality evidence for their efficacy [143]. Moreover, there is some evidence to support the idea that Cannabis is associated with an increased risk of adverse mental health effects. However, that evidence is generally quite weak as the studies are of low quality, have limited participant numbers, short study durations, a wide variety of cannabinoid preparations and doses, and a frequently, a high rate of bias.

Conclusions in studies into reducing opioid doses in the management of chronic pain, where some trials have shown clinical benefits, are sometimes not completely reliable as they inadequately report dose changes and have mixed results in analgesic effects [144]. Recent analysis has found no evidence to suggest that Cannabis can exert an opioid-sparing effect [145]. 

Concerning the treatment of inflammatory bowel diseases with cannabinoids, preclinical evidence has indicated that CBD protects against intestinal inflammation (reviewed in [146]). However, GW Pharmaceuticals, who completed a phase IIa pilot study in 2014 did not list CBD for the treatment of ulcerative colitis on its development pipeline [147]. Only products from Vitality Biopharma (cannabinoid prodrugs) seem to be designed for a targeted approach to the gut. Nevertheless, there is global demand for larger clinical trials to be conducted to reveal whether treatment with cannabinoids or their derivatives can provide benefits to inflammatory bowel disease patients.

The impact of cannabinoids on patient-reported outcomes, such as health-related quality of life, has recently been analysed by Goldenberg in a systematic review [148]. Once again, results were disappointing, although there were some small improvements in health-related quality of life for some patients with pain, multiple sclerosis and inflammatory bowel disease. However, reduced effects were observed in some patients with HIV, leading the authors to conclude that the evidence for the effects of cannabinoids on health-related quality of life is inconclusive. The information that is currently available in the reports of reliable randomized controlled trials is clearly limited, although there are increasing reports of considerable subjective effects (pain treatment). 

Other systematic reviews have also described harm caused and some commonly reported adverse effects. Cannabis seems to be associated with harm to the central nervous system and the gastro-intestinal system [142,149].

It would therefore appear that the clinical evidence collated to date is confounded by a number of factors, including studies with mixed patient populations, use of different cannabinoid preparations and in various formulations, and wide dosing ranges.

Cannabis-derivative-based medicines may be able to enrich the drug treatment arsenal for chronic pain and inflammation conditions, although this is very much open to debate at the moment. CBD, unlike THC, is not considered an abused drug and several industries are involved in the production of CBD as an active pharmaceutical ingredient with the highest quality standard. It is relevant, and expected, that regulatory agencies, other than the Medications Health Care Products Regulation Agency, will evaluate and approve CBD as a medicine after a careful study of quality, safety and efficacy data [13]. While medicinal cannabis has already entered mainstream medicine in many countries, particular care should be taken in a period in which the on-line availability of a variety of CBD-based products for therapeutic purposes, such as oils, tinctures and vapours, has rapidly expanded and, along with it, an increase in potential health risks for patients/consumers may be expected. 

## 5. Concluding Remarks

Cannabinoids and endocannabinoids are a hot topic in the fields of chemical and biomedical research with more than 1000 articles being published per year and the trend is for that to increase. Furthermore, research into cannabinoid delivery systems is growing and a plethora of patents have shown interest in the companies working in this field, especially when it comes to local/transdermal administration. Combining formulations may provide an opportunity to produce rapid systemic effects and long-term outcomes (e.g., analgesia). This could be achieved with intranasal cannabinoid sprays used as a low-dose adjuvant to patches in order to aid rapid absorption for systemic effects. Interesting and promising transdermal administration results can also be found in the use of terpenes (from the same source) as CBD and THC penetration enhancers, and thus improve the effectiveness of the therapeutic components. This, once again, highlights the role that quality plays in defining the composition, dosage and related safety of the components extracted from cannabis. 

It is expected that recent developments in pharmacological, pharmaceutical and technological sciences will result in new therapeutic strategies using both known cannabinoids for new therapeutic strategies as well as cannabinoid synthetic derivatives. 

Nanotechnology is indeed a promising approach that may bring cannabinoids closer to clinical use (the SEDDS approach is a fine example), and administration via both the oral and pulmonary routes. Furthermore, it is at an early stage the use of well-known advanced nanomaterials in cannabinoid delivery (e.g., carbon nanotubes). Nevertheless, additional evaluation is required if the cost effectiveness and long-term safety of nano-delivery systems is to be improved. 

## Figures and Tables

**Table 1:** Currently available dosage forms for cannabinoids and their innovative delivery systems.

Administration Route	Name	Drug	Delivery System/ Dosage Form	Disease	Application	Development Stage	References
Oral	Dronabinol	THC	Solid	HIV, chemotherapy	Anorexia, nausea	Market	[56]
Oral	Nabilone	THC analogue	Solid	Chemotherapy, chronic pain	Nausea, pain	Market	[59,60]
Oral	Epidiolex	CBD	Liquid	Lennox-Gastaud and Dravet syndromes	Epilepsy	Market	[62,63,64]
Oral		CBD	Solid	Crohn’s disease, GVHD		Clinical trials	[66]
Oral		THC	SEDDS		Improving dissolution, stability	Preclinical	[69,70,71]
Oral		THC-glycosides	Prodrugs	Drug-resistant inflammatory bowel disease	Inflammation	Clinical trials	[72,73]
Oromucosal	Nabiximols	THC CBD 1:1	Spray	Multiple sclerosis	Spasticity	Market	[75,78]
Oromucosal				Cancer	Pain	Clinical trials	[77]
Oromucosal		CBD	Powder			Formulation study	[79]
Oromucosal		THC CBD 1:1	Chewing-gum	Several potential diseases	Pain, spasticity, dementia etc.	Preclinical	[80]
Intranasal		CBD	Liquid formulations		Bioavailability study	Preclinical	[82]
Pulmonary		CBD	Solid/liquid			Formulation study	[86]
Pulmonary			Powder metered-dose inhaler		Bioavailability study	Clinical trials	[87]
Transdermal		Phytocannabinoids		Induced dermatitis	Inflammation	Preclinical	[92]
Transdermal		CBD	Gel	Arthritis	Inflammation	Preclinical	[93]
Transdermal		CBD	Ethosomes	Oedema	Inflammation	Preclinical	[95]
Transdermal		CBD	Gel	Epilepsy, osteoarthritis, fragile-X syndrome		Clinical trials	[96,97,98]
Transdermal		CBD	Oil, spray, cream	Epidermiolysis bullosa	Pain, blistering	Clinical treatment	[100]
Transdermal		CBD	Patch			Formulation study	[112]
Transdermal		CBD + hyaluronic acid	Gel	Pain, wound management		Formulation study	[105]
Transdermal		CBD+ argan oil		Rheumatic diseases	Inflammation	Formulation study	[107]
Transdermal		CBD+boswellic acid			Inflammation	Formulation study	[108]
Topical ocular		THC analogue	Prodrugs	Glaucoma	Reduce intraocular pressure	Formulation study	[111]

THC, Δ^9^-tetrahydrocannabinol; CBD, cannabidiol; GVHD, graft-versus-host disease; SEDDS, Self-emulsifying drug delivery systems.

**Table 2:** Nanosized cannabinoid delivery systems.

Type		Constituents	Drug	Size (nm)	Encapsulation Efficiency	Application	Development Stage	References
**Lipid-based**	liposomes	DPPC, cholesterol	THC	300–500	0.3 mg/mL	i.v.	Pharmacokinetics	[117]
	micelles	PC, PE plus phospholipids	Terpenes, hemp oil		n.d.		Stability evaluations	[118]
	micelles	Polyethoxylated castor oil, glycerol	Cannabis oil	100	n.d.	oromucosal	Clinical trials	[119,120]
	NCL	tristearin/tricaprylin 2:1	Cannabinoids	100	high		Formulation study	[122]
	NCL	Cetyl palmitate or glyceryl dibehenate	THC	200	n.d.	nasal	Preclinical studies	[123]
	NCL	Glyceryl dibehenate or glyceryl palmitostearate	CB-13	120	99%	oral	Preclinical studies	[125]
	PNL	PTL401	THC CBD 1:1	<50	99%	oral	Preclinical studies	[130]
	PNL	PTL401	Plus piperine	<50	99%	oral	Clinical trials	[131,132]
	Nanoemulsions					rectal/vaginal	n.d.	[133]
**Polymeric-based**	PLGA	plus coating agents	CB-13	253–344	85%	oral	Preclinical studies	[137]
	PLGA	plus coating agents	THC	290–800	96%	oral	Preclinical studies	[138]
	PCL		CBD	2000–5000	100%	locoregional	Preclinical studies	[139]

NCL, nanostructured lipid carrier; PNL, pro-nano-liposphere; PLGA, poly(lactic-co-glycolic acid); PCL, Poly-ε-caprolactone; PC, phosphatidylcholine; PE, phosphatidylethanolamine; EE = encapsulation efficiency calculated as (total drug added-free non-entrapped drug) divided by the total drug added; PLT401 is a proprietary formulation containing polysorbate 20, sorbitan monooleate 80, polyoxyethylene hydrogenated castor oil 40, glyceryl tridecanoate, lecithin and ethyl lactate; n.d., not defined.
